# Big five personality, anxiety sensitivity, and Turkish L2 speaking anxiety in international students: a mediation model

**DOI:** 10.3389/fpsyg.2026.1806796

**Published:** 2026-07-29

**Authors:** Ibrahim Erdoğan Yayla, Kübra Dombak, Sedat Maden, Ibrahim Arpaci, Mustafa Baloglu

**Affiliations:** 1 Faculty of Education, Guidance and Psychological Counseling, Bayburt University, Bayburt, Türkiye; 2 Faculty of Education, Guidance and Psychological Counseling, Sakarya University, Sakarya, Türkiye; 3 Department of Turkish Education, Faculty of Education, Bayburt University, Bayburt, Türkiye; 4 Department of Computer Engineering, Faculty of Engineering, Bursa Uludag University, Bursa, Türkiye; 5 Department of Computer Science and Engineering, College of Informatics, Korea University, Seoul, Republic of Korea; 6 Department of Special and Gifted Education, College of Education, United Arab Emirates University, Abu Dhabi, United Arab Emirates

**Keywords:** anxiety sensitivity, big five, foreign language, L2 speaking anxiety, mediation, personality

## Abstract

**Introduction:**

Second language (L2) speaking anxiety is a significant affective variable that can limit the communication processes and academic experiences of foreign language learners. Although interest in the relationship between personality traits and speaking anxiety has increased in recent years, the psychological processes through which this relationship manifests have not been sufficiently investigated.

**Method:**

The mediating role of anxiety sensitivity in the relationship between the Five-Factor Model of personality traits and L2 speaking anxiety was examined. The research is a cross-sectional, quantitative study conducted with international university students learning Turkish as a second language (*N* = 286; 76.6% male). The data were analyzed using bootstrap-based mediation analyses.

**Result:**

We found significant negative correlations between conscientiousness, agreeableness, extroversion, and openness to experience and L2 speaking anxiety, and a significant positive correlation with neuroticism. Mediation analyses showed that anxiety sensitivity had significant indirect effects in the relationships between all five personality dimensions and L2 speaking anxiety.

**Discussion:**

The findings suggest that both personality traits and anxiety sensitivity should be considered when interpreting Turkish L2 speaking anxiety.

## Introduction

Second-language (L2) speaking anxiety is one of the key affective variables thought to influence individuals' willingness to communicate and their oral participation during the language-learning process (Lee and Chiu, 2023). L2 speaking anxiety is defined as the state of tension experienced by an individual due to concerns about making mistakes, being misunderstood, or being judged negatively whilst speaking (Okyar, 2023). Studies indicate that speaking anxiety is associated with language proficiency and characteristics of the learning environment (Akter, 2024; Kayhan, 2025). However, recent research suggests that L2 speaking anxiety may be linked not only to linguistic processes but also to individuals' personality traits and psychological predispositions regarding anxiety (Gao, 2022). Therefore, examining speaking anxiety within the context of individual differences may contribute to a broader understanding of the emotional experiences involved in second-language learning.

### The relationship between the personality traits and L2 speaking anxiety

One of the most decisive psychological components of individual differences in foreign language learning is personality traits (Chen et al., 2022). Personality can be defined as the set of characteristics a person is born with and acquires throughout their life that distinguish them from other individuals; it is a consistent pattern of behavior (Roberts and Yoon, 2022). Among the theories developed to explain personality structure, the Five-Factor Personality Model is one of the prominent approaches. Also referred to as the OCEAN model, this theoretical framework examines individuals' personality traits under five basic dimensions: agreeableness, conscientiousness, extraversion, neuroticism, and openness to experience (Chen et al., 2022; Li and Xu, 2025). The following behavioral traits characterize these dimensions:

- Extraversion: approachable, energetic, cheerful, sensation-seeking, dominant.
- Agreeableness: humble, cooperative, sincere, understanding.
- Conscientiousness: systematic, determined, success-oriented, careful, planned.
- Neuroticism: tendency to experience emotional instability, including worry, tension, insecurity, heightened emotional reactivity to criticism and negative evaluation, and difficulty managing stressful situations.
- Openness to experience: creative, analytical, curious, and receptive to new ideas, different viewpoints, and aesthetic or emotional experiences.

Beyond explaining individuals' behavioral patterns, the Five-Factor Personality Model is also considered a significant explanatory variable for understanding learning styles, emotional responses, and social relationships (Karataş and Arpaci, 2026; Arpaci et al., 2025; Qiao, 2024). In this context, personality traits are among the fundamental elements shaping an individual's learning process and performance, especially in areas where emotional processes play a significant role, such as language learning and communication skills (Roberts and Yoon, 2022). It is stated that emotional responses, particularly speaking anxiety, are closely related to an individual's personality structure (Chen et al., 2022; Erzhanova et al., 2024). Current studies show that personality traits significantly influence second language (L2) speaking anxiety. Erzhanova et al. (2024) showed that individual personality traits are strong determinants of foreign language classroom anxiety. That personality plays a decisive role in students' communicative self-confidence and anxiety regulation. Similarly, research examining the indirect effects of personality traits on anxiety, stress, and academic performance in foreign language learning emphasizes that these relationships operate through variables such as emotional stability, self-confidence, and willingness to communicate (Gao, 2022). Accordingly, personality structure is considered an important determinant of students' attitudes toward classroom interactions, their anxiety about making mistakes, and their expectations of social acceptance (Akaraphattanawong et al., 2024).

Studies examining the relationship between personality traits and second-language speaking anxiety have found that stress levels experienced during speech are related to personality structure (Chen et al., 2022; Lindberg et al., 2023). Furthermore, it is stated that individuals' general personality tendencies strongly predict levels of shyness, risk-taking behaviors, and tolerance for making mistakes that emerge during language performance (Barua, 2022). In a separate study of university students, it was found that success in foreign language classes is closely linked to language anxiety and personality traits; individuals with low anxiety levels and balanced personalities achieved higher scores on language exams and speaking performance (Gao, 2022). Similarly, another study investigating how personality traits directly and indirectly impact foreign language anxiety revealed that self-efficacy mediates this relationship. Individuals with high levels of extraversion and openness to experience tend to have strong self-efficacy perceptions and, therefore, lower anxiety levels; in contrast, those with high neurotic tendencies display significantly higher anxiety (Qin and Li, 2024). All these studies highlight that personality structure is a key psychological factor influencing L2 speaking anxiety. Specifically, personality-based factors such as social interaction orientation, self-control, and emotional stability directly affect the tension experienced when speaking a foreign language and perceptions of self-efficacy (Wu et al., 2024). In this context, personality structure not only shapes how individuals behave during language learning but also impacts their anxiety levels in the learning environment.

In summary, whilst the findings suggest that personality traits may be associated with L2 speaking anxiety, it remains unclear through which psychological processes this relationship is mediated. In this regard, individuals' perceptions of anxiety-provoking situations and how they interpret the physical or cognitive symptoms that arise in such situations may be of particular significance. The literature indicates that personality traits may be associated with individuals' levels of anxiety sensitivity; this sensitivity, in turn, can be considered alongside anxiety experiences encountered in performance-demanding communicative situations (Qin and Li, 2024; He and Wang, 2024). Building on this, it seems meaningful to examine the relationship between personality traits and L2 speaking anxiety not only through direct relationships but also within the context of individual psychological tendencies such as anxiety sensitivity.

### The mediating role of anxiety sensitivity

During the process of learning a second language, individuals' personality traits, affective responses, and cognitive tendencies directly influence their learning performance (Papi and Khajavy, 2023). In this process, anxiety sensitivity is an important psychological variable that refers to an individual's perception, interpretation, and emotional response to anxiety symptoms (Daymiel et al., 2022). Individuals with high anxiety sensitivity may experience higher levels of L2 speaking anxiety by perceiving the possibility of making mistakes or being negatively evaluated during speech as a threat (Zhao, 2024). Recent research also supports this relationship. Daymiel et al. (2022) found that foreign language speaking anxiety significantly reduces students' vocabulary, pronunciation, and comprehension performance, and that high anxiety levels lead to avoidance of communication (Daymiel et al., 2022). Similarly, Ady and Mardiah (2024) reported that foreign-language speaking anxiety is one of the most prominent affective variables that negatively predict students' self-confidence and willingness to participate. Additionally, Nastiti (2023) revealed that L2 speaking anxiety is not limited to classroom situations; individuals experience higher levels of anxiety when communicating in social settings, especially with native speakers. This result demonstrates that anxiety sensitivity is not merely a cognitively based predisposition but also a stress response that emerges in social interaction settings. Furthermore, a comprehensive review study reveals that foreign language anxiety exhibits a multidimensional structure and is closely related to learners' personality traits, prior experiences, and emotional sensitivities (Zheng et al., 2024).

Personality structure is considered among the individual characteristics related to an individual's anxiety sensitivity (Kiliçaslan et al., 2022; Wu et al., 2024). It is noted that individuals with high levels of neuroticism tend to exhibit higher anxiety sensitivity, and this tendency is related to second language speaking anxiety in the language learning process (Liu, 2024). In this context, it is noteworthy that individuals perceive their mistakes in stressful learning environments as more threatening and evaluate their communication performance more narrowly. Similarly, relevant research indicates that neuroticism is associated with sub-dimensions of foreign language anxiety such as fear of negative evaluation, communication anxiety, and test anxiety; conversely, extraversion and openness to experience are associated with lower levels of anxiety experience (Erzhanova et al., 2024). Furthermore, in a study conducted by Smyth et al. (2021) with undergraduate students, the relationship between foreign language anxiety and academic performance was examined alongside personality traits; neuroticism and extraversion, in particular, were reported as prominent variables in this pattern (Gao, 2022). This finding suggests that personality not only functions as a trait that can be assessed alongside emotional tendencies but also acts as a variable associated with learning performance in conjunction with anxiety sensitivity. Furthermore, Nabella and Umami (2023) noted that higher levels of introversion in students were associated with higher levels of anxiety during speech performance. In contrast, extroverted individuals were associated with higher levels of self-esteem and lower communication anxiety (Nabella and Umami, 2023).

The research findings suggest that anxiety sensitivity can be considered a mediating variable in the relationship between personality traits and language anxiety. Qin and Li (2024) found that in the relationship between foreign language anxiety and personality traits, anxiety sensitivity and self-efficacy together have a significant mediating effect. The study revealed that students with high neuroticism levels exhibited low self-efficacy and high anxiety sensitivity, which in turn increased L2 speaking anxiety. Similarly, a study indicated that agreeableness, as a personality trait, is associated with subjective perceptions of foreign language skills; however, this relationship is attenuated by language anxiety (Piechurska-Kuciel et al., 2024). The findings demonstrate that high anxiety levels suppress the positive effects of personality traits on perceived communicative competence. Furthermore, another study conducted by He and Wang (2024) found that awareness and anxiety sensitivity play a significant mediating role in the relationship between state and trait anxiety levels and foreign language anxiety. The research reported that people with high anxiety sensitivity experienced more stress in foreign language learning and had lower speaking performance (He and Wang, 2024). These findings suggest that anxiety sensitivity functions as an important psychological bridge in the relationship between personality traits and L2 speaking anxiety. When the relationships between personality traits and anxiety sensitivity are considered together, this sensitivity also determines the level of anxiety observed in language performance. In this context, it can be proposed that in second language learners, personality structure affects L2 speaking anxiety not directly, but through anxiety sensitivity.

### Present study

In an increasingly globalized world, dynamics such as migration, education, and intercultural interaction are making second language learning increasingly important. However, one of the key challenges faced by learners of a foreign language is speaking anxiety, which plays a significant role in the development of communication skills. Second-language speaking anxiety can hinder individuals' ability to use the language effectively, participate in communication, and express themselves with confidence (Baissane, 2023). The literature indicates that individual differences, such as personality traits, self-efficacy, motivation and emotional intelligence, are key variables in understanding L2 speaking anxiety (Erzhanova et al., 2024; He and Wang, 2024). In particular, personality dimensions within the Five-Factor Model of Personality are associated with foreign-language speaking anxiety. Research indicates that high levels of neuroticism are associated with higher levels of speaking anxiety, whilst traits such as openness to experience and extraversion are associated with lower levels of anxiety (Erzhanova et al., 2024; Vural, 2019).

Studies conducted with individuals learning Turkish as a second language also indicate that speaking anxiety is a significant affective variable within this group of learners. In particular, anxiety regarding speaking skills has been examined in relation to participation in the communication process, speaking performance, and speaking self-efficacy (Okyar, 2023; Uzun, 2026). However, it is observed that there is a limited number of studies examining speaking anxiety in international students learning Turkish as a second language in conjunction with individual psychological characteristics such as personality traits and anxiety sensitivity (Zhang and Tan, 2024).

However, the mediating role of anxiety sensitivity in the relationship between personality traits and L2 speaking anxiety has not been sufficiently explored in the literature. An individual's cognitive and emotional responses to anxiety during the language-learning process are among the variables considered when understanding how speaking experiences are perceived (Chen et al., 2024). In this context, examining relatively enduring personality traits alongside individual sensitivity to anxiety may help understand the affective aspects of the language-learning process. This study examines the mediating role of anxiety sensitivity in the relationship between the Five-Factor Model of Personality and L2 speaking anxiety. It is expected that the study will contribute to the relevant literature by integrating personality psychology with the affective processes involved in second language learning.

**Figure 1:**
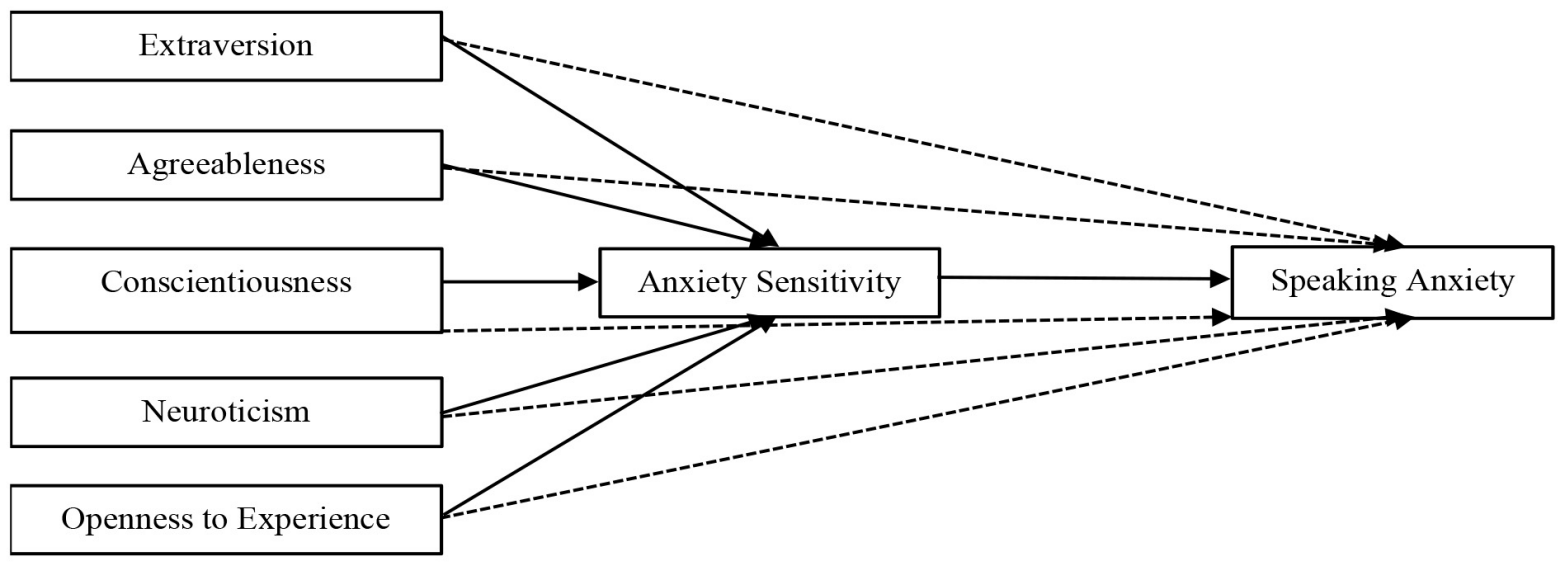
Hypothetical model.

In light of current theoretical explanations and empirical findings, it appears that personality traits may be associated with individuals' anxiety levels during speaking. However, it remains unclear how this relationship varies across all personality dimensions, nor what role anxiety sensitivity plays in this relationship. In the context of international students learning Turkish as a second language, there is a need for a more comprehensive examination of the relationship between personality traits and speaking anxiety. In this context, this study aimed to investigate the relationships between Big Five personality (BFP) traits, anxiety sensitivity, and anxiety about speaking Turkish as a second language among international students. Furthermore, the study aimed to examine the mediating role of anxiety sensitivity in the relationship between BFP traits and anxiety about speaking Turkish as a second language. The following hypotheses were tested in the present research:

H1. Big Five personality traits are significantly associated with Turkish L2 speaking anxiety.
H2. Big Five personality traits are significantly associated with anxiety sensitivity.
H3. Anxiety sensitivity is significantly associated with Turkish L2 speaking anxiety.
H4. Anxiety sensitivity mediates the relationships between Big Five personality traits and Turkish L2 speaking anxiety (Figure 1).

## Method

### Participants

Participants were foreign university students studying Turkish as a second language (*N* = 286; 76.6% male). Their ages ranged from 18 to 48, with a mean age of 19.9 (SD = 3.9). The sample comprises international university students enrolled in the Turkish Language Teaching Centers of three state universities in various provincial capitals across Turkey. Participants took part in the study voluntarily, and convenience sampling was used during data collection. The link to the online survey was shared with the participants via the relevant student groups. To evaluate whether the sample size was adequate for the mediation analyses, a *post hoc* power analysis was conducted at α = 0.05 using G^*^Power. The results showed that even for a small effect size (*f*^2^ = 0.02), the statistical power for [*N* = 286] exceeded 0.90, whereas the power for medium (*f*^2^ = 0.15) and large (*f*^2^ = 0.35) effect sizes was approximately 1.00. These findings indicate that the sample size is statistically sufficient to examine the mediation model tested in the study.

### Instruments

#### Anxiety sensitivity index-3

Developed to determine individuals' levels of sensitivity to anxiety symptoms. The scale [Original version: Taylor et al. (2007); Turkish version: Mantar et al. (2010)] consists of 18 items and is a five-point Likert scale. It has three subscales: physical, cognitive, and social. The internal consistency coefficients of the scale are 0.89 for “physical symptoms,” 0.88 for “cognitive symptoms,” and 0.82 for “social symptoms.” The factor analysis conducted to assess construct validity revealed that the scale's three-dimensional structure was preserved, with item loadings ranging from 0.40 to 0.79 and a total variance explained of 61.72% (Mantar et al., 2010). In the reliability analyses conducted on data obtained in this study, the scale's Cronbach's α was 0.90, and McDonald's ω was 0.90.

#### The five-factor personality scale

The scale was developed to assess individuals' personality traits across five fundamental dimensions: extraversion, neuroticism, openness to experience, agreeableness, and conscientiousness. The scale [Original version: Rammstedt and John (2007); Turkish version: Horzum et al. (2017)] consists of 10 items and is a five-point Likert scale. As a result of the EFA, the scale retained its five-factor structure and explained 88.4% of the total variance. The CFA results (χ^2^/df = 1.84, SRMR = 0.03, CFI = 0.98, GFI = 0.96, RMSEA = 0.062) indicated that the model fit well, and the convergent and discriminant validity analyses supported the scale's construct validity. Cronbach's alpha values for the subscales ranged from 0.81 to 0.90. In this study's sample, Cronbach's alpha coefficients for the sub-dimensions ranged from 0.79 to 0.85.

#### The speaking anxiety scale for foreigners learning Turkish

The scale was developed to determine the levels of speaking anxiety experienced by individuals learning Turkish as a second language. The scale [Original version: Woodrow (2006); Turkish version: Melanlioglu and Demir (2013)] comprises 12 items and uses a 5-point Likert format. It has two subscales: “out-of-class speaking anxiety” and “in-class speaking anxiety.” The EFA revealed that the scale's two-factor structure was preserved, with item factor loadings ranging from 0.50 to 0.79 and explaining 61% of the total variance. The CFA results (CFI = 0.97, NFI = 0.93, IFI = 0.97, RMSEA = 0.065) indicated that the model provided a good fit. The internal consistency coefficients for the scale were 0.90 and 0.93 for the subscales, and 0.91 for the total scale. In the reliability analyses conducted using the data obtained in this study, the scale's Cronbach's α coefficient was 0.94, and McDonald's ω coefficient was 0.94.

### Procedure

The research was conducted with the necessary approval from the ethics committee of the relevant university (Ethics Committee Approval No: 2025/262, Date: May 16, 2025). Following ethical approval, an online survey form was prepared and presented to participants via the internet. At the beginning of the survey, all participants were informed about the research aim, the principles of confidentiality, and the voluntary nature of their participation, after which they provided electronic consent. The study included individuals aged 18 and over who were learning Turkish as a second language at university Turkish Language Teaching Centers, were literate in Turkish, and completed the questionnaire in full. All assessment tools were administered in Turkish. Participants' proficiency in Turkish was not assessed via a separate language test. However, all participants were students who were continuing to receive Turkish language instruction during the data collection process. Participants under 18, those who left the survey incomplete, and those who provided inconsistent responses (e.g., “selecting the same option for all items”) were excluded from the dataset. Data were collected over approximately 4 weeks.

### Data analysis

This study examined the mediating role of anxiety sensitivity in the relationship between Big Five personality traits and speaking anxiety among international students learning Turkish as a second language. Before beginning the analyses, the assumptions of parametric tests, namely normality, linearity, and multicollinearity, were tested, and the findings are presented in Table 1. Harman's one-factor test was applied to examine potential common-method bias before commencing data analyses (Kock, 2015). The results of the unrotated principal component analysis indicated that the first component explained 29.49% of the total variance. The fact that the proportion of variance explained by the first component remained below 50% was interpreted as indicating that systematic bias from common-method factors was not dominant in the dataset. Subsequently, Pearson's product-moment correlation analysis was performed to determine the strength and direction of the correlations between the study variables, and the results are presented in Table 2. In the next stage, Hayes' (2022) PROCESS macro (Model 4) was used to test the mediating role of anxiety sensitivity in the relationship between the five-factor personality structure and speaking anxiety. The Process Macro add-on was used with SPSS 31 to analyze the data.

**Table 1:** Descriptive statistics, linearity, normality, and multicollinearity.

Variables	*N*	M	SD	Skew.	Kurt.	Tol.	VIF	CI
1. Speaking anxiety	286	27.04	11.70	0.55	−0.10	–	–	1.00
2. Anxiety sensitivity	286	26.56	14.13	0.27	0.03	0.83	1.21	4.85
3. Extraversion	286	6.40	2.23	−0.08	−1.15	0.81	1.24	7.34
4. Agreeableness	286	6.72	1.73	−0.01	−0.46	0.80	1.25	11.10
5. Conscientiousness	286	6.89	1.58	−0.26	0.36	0.90	1.11	12.22
6. Neuroticism	286	5.81	2.03	0.13	−0.95	0.82	1.22	9.13
7. Openness to experience	286	6.68	2.08	−0.20	−0.71	0.83	1.20	25.13

**Table 2:** Pearson intercorrelations of the variables.

Variables	1	2	3	4	5	6	7
1. Speaking anxiety	1						
2. Anxiety sensitivity	0.58^**^	1					
3. Extraversion	−0.32^**^	−0.24^**^	1				
4. Agreeableness	−0.23^**^	−0.22^**^	0.36^**^	1			
5. Conscientiousness	−0.28^**^	−0.23^**^	0.15^**^	0.12^**^	1		
6. Neuroticism	0.33^**^	0.21^**^	−0.30^**^	−0.34^**^	−0.13^**^	1	
7. Openness to experience	−0.45^**^	−0.33^**^	0.19^**^	0.15^**^	0.25^**^	−0.23^**^	1

^**^p < 0.01.

## Results

Descriptive statistics for the variables examined in this study, along with indicators of linear relationship, normality, and multicollinearity, are presented in Table 1. The mean for speaking anxiety was *M* = 27.04 (SD = 11.70), and the mean for anxiety sensitivity was *M* = 26.56 (SD = 14.13). The mean (*M*) and standard deviation (SD) values obtained for the five-factor personality dimensions of conscientiousness, agreeableness, neuroticism, extraversion, and openness to experience are also presented in Table 1.

The skewness and kurtosis coefficients for the variables range from −0.26 to 0.55 and from −1.15 to 0.95, respectively. These values are consistent with the normal distribution limits accepted for applied research. When examining multicollinearity indicators, tolerance values range from 0.81 to 0.90, and VIF values range from 1.11 to 1.25. The literature indicates that tolerance values falling below 0.10 and VIF values exceeding 10 pose a risk (Field, 2024). Since the values obtained in this study fall outside these thresholds, there is no significant multicollinearity in the model.

Table 2 shows the Pearson correlations between variables. A positively significant relationship was observed between speaking anxiety and anxiety sensitivity (*r* = 0.58, *p* < 0.01) and neuroticism (*r* = 0.33, *p* < 0.01). In contrast, extraversion (*r* = −0.32, *p* < 0.01), agreeableness (*r* = −0.23, *p* < 0.01), conscientiousness (*r* = −0.28, *p* < 0.01), and openness to experience (*r* = −0.45, *p* < 0.01) are negatively related to speaking anxiety.

Table 3 presents the regression results for predicting speaking anxiety from extraversion. Extraversion was found to have a negative and significant impact on speaking anxiety (β = −0.32, *p* < 0.001). The model is significant, *F*(1, 284) = 32.35, *p* < 0.001, and explains 10% of the total variance (*R*^2^ = 0.10).

**Table 3:** Regression coefficients, standard errors, and significance tests for the regression model.

Predictor	Coeff.	*SE*	*p*	*F*	*R^2^*
Constant	37.79	2.00	< 0.001		
Extraversion	−0.32	0.29	< 0.001	32.35	0.10

Table 4 presents the findings from the mediation analysis. Extraversion significantly predicts anxiety sensitivity, and this relationship is negative (*a* = −0.24, *p* < 0.001). Anxiety sensitivity is positively and significantly related to speaking anxiety (b = 0.54, *p* < 0.001). The direct relationship between extraversion and speaking anxiety (*c*) is significant in the first model (β = −0.32, *p* < 0.001), but the effect size decreases when anxiety sensitivity is added to the model (*c*′ = −0.19, *p* < 0.001). This decrease supports the partial mediating effect.

**Table 4:** The mediating role of anxiety sensitivity between extraversion and speaking anxiety.

Model	Anxiety sensitivity (*M*)			Speaking anxiety (Y)		
	β	SE	*t*	β	SE	*t*
Extraversion (X) *a*	−0.24	0.36	−4.11	*c'* −0.19	0.25	−3.98
Anxiety sensitivity (*M*)	–	–	–	*b* 0.54	0.04	11.06
Constant	36.18	2.48	14.61	21.74	2.22	9.81
	*R*^2^ = 0.06, *F*_(1, 284)_ = 16.94, *p* < 0.001			*R*^2^ = 0.38, *F*_(2, 283)_ = 84.21, *p* < 0.001		
**Bootstrap results for indirect effects**	β			**Boot SE**	**BootLLCI**	**BootULCI**
Indirect effect of anxiety sensitivity	−0.13			0.04	−0.20	−0.06

**Figure 2:**
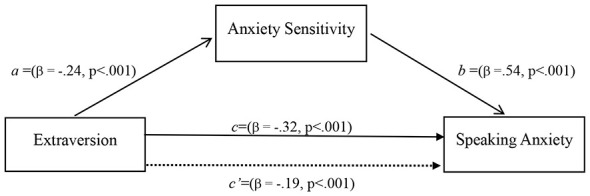
The mediating model of anxiety sensitivity in the relationship between extraversion and speaking anxiety.

The Bootstrap analysis revealed that extraversion had a statistically significant indirect effect on speaking anxiety (β = −0.13, Boot SE = 0.04, 95% CI [−0.20, −0.06]). The fact that the confidence interval (CI) does not include zero reveals that the mediating effect is significant. Figure 2 summarizes the partial mediation model obtained.

Table 5 presents the regression results for predicting speaking anxiety from agreeableness. Agreeableness was found to have a negative and significant impact on speaking anxiety (β = −0.23, *p* < 0.001). The model is significant, *F*(1, 284) = 15.93, *p* < 0.001, and explains 5% of the total variance (*R*^2^ = 0.05).

**Table 5:** Regression coefficients, standard errors, and significance tests for the regression model.

Predictor	Coeff.	*SE*	*p*	*F*	*R^2^*
Constant	37.50	2.70	< 0.001		
Agreeableness	−0.23	0.39	< 0.001	15.93	0.05

Table 6 presents the findings from the mediation analysis. Agreeableness significantly predicts anxiety sensitivity, and this relationship is negative (*a* = −0.22, *p* < 0.001). Anxiety sensitivity is positively and significantly related to speaking anxiety (b = 0.56, *p* < 0.001). The direct relationship between agreeableness and speaking anxiety (*c*) is significant in the first model (β = −0.23, *p* < 0.001), but the effect size decreases when anxiety sensitivity is added to the model (*c*′ = −0.11, *p* < 0.001). This decrease supports the partial mediating effect.

**Table 6:** The mediating role of anxiety sensitivity between agreeableness and speaking anxiety.

Model	Anxiety sensitivity (*M*)			Speaking anxiety (Y)		
	β	SE	*t*	β	SE	*t*
Agreeableness (X) *a*	−0.22	0.47	−3.76	*c'* −0.11	0.33	−2.22
Anxiety sensitivity (*M*)	–	–	–	*b* 0.56	0.04	11.35
Constant	38.47	3.28	11.74	19.73	2.74	7.21
	*R*^2^ = 0.05, *F*_(1, 284)_ = 14.11, *p* < 0.001			*R*^2^ = 0.35, *F*_(2, 283)_ = 75.99, *p* < 0.001		
**Bootstrap results for indirect effects**	β			**Boot SE**	**BootLLCI**	**BootULCI**
Indirect effect of anxiety sensitivity	−0.12			0.04	−0.19	−0.05

**Figure 3:**
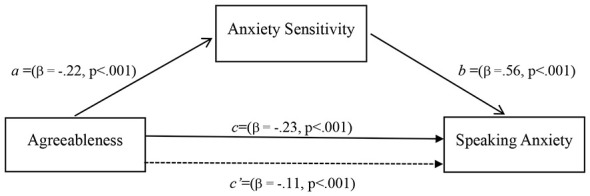
The mediating model of anxiety sensitivity in the relationship between agreeableness and speaking anxiety.

The Bootstrap analysis revealed that agreeableness had a statistically significant indirect effect on speaking anxiety (β = −0.12, Boot SE = 0.04, 95% CI [−0.19, −0.05]). The fact that the confidence interval (CI) does not include zero indicates that the mediating effect is significant. Figure 3 summarizes the partial mediation model obtained.

Table 7 presents the regression results for predicting speaking anxiety from conscientiousness. Conscientiousness was found to have a negative and significant effect on speaking anxiety (β = −0.28, *p* < 0.001). The model is significant, *F*(1, 284) = 24.07, *p* < 0.001, and explains 8% of the total variance (*R*^2^ = 0.08).

**Table 7:** Regression coefficients, standard errors, and significance tests for the regression model.

Predictor	Coeff.	*SE*	*p*	*F*	*R^2^*
Constant	41.33	2.99	< 0.001		
Conscientiousness	−0.28	0.42	< 0.001	24.07	0.08

Table 8 presents the findings from the mediation analysis. Conscientiousness significantly predicts anxiety sensitivity, and this relationship is negative (*a* = −0.23, *p* < 0.001). Anxiety sensitivity is positively and significantly related to speaking anxiety (b = 0.55, *p* < 0.001). The direct relationship between conscientiousness and speaking anxiety (*c*) is significant in the first model (β = −0.28, *p* < 0.001), but the effect size decreases when anxiety sensitivity is added to the model (*c*′ = −0.16, *p* < 0.001). This decrease supports the partial mediating effect.

**Table 8:** The mediating role of anxiety sensitivity between conscientiousness and speaking anxiety.

Model	Anxiety sensitivity (*M*)			Speaking anxiety (Y)		
	β	SE	*t*	β	SE	*t*
Conscientiousness (X) *a*	−0.23	0.52	−3.92	*c'* −0.16	0.36	−3.19
Anxiety sensitivity (*M*)	–	–	–	*b* 0.55	0.04	11.20
Constant	40.53	3.66	11.08	23.00	2.98	7.72
	*R*^2^ = 0.05, *F*_(1, 284)_ = 15.34, *p* < 0.001			*R*^2^ = 0.36, *F*_(2, 283)_ = 79.98, *p* < 0.001		
**Bootstrap results for indirect effects**	β			**Boot SE**	**BootLLCI**	**BootULCI**
Indirect effect of anxiety sensitivity	−0.12			0.03	−0.19	−0.06

**Figure 4:**
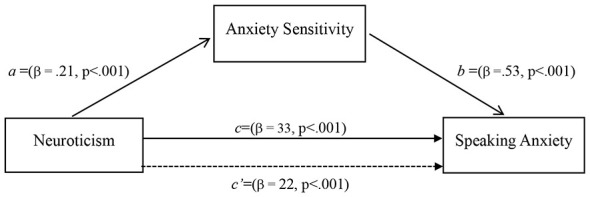
The mediating model of anxiety sensitivity in the relationship between conscientiousness and speaking anxiety.

The bootstrap analysis revealed that conscientiousness had a statistically significant indirect effect on speaking anxiety (β = −0.12, Boot SE = 0.03, 95% CI [−0.19, −0.06]). The fact that the confidence interval (CI) does not include zero indicates that the partial mediating effect is significant. Figure 4 summarizes the obtained mediation model.

Table 9 presents the regression results regarding the prediction of speaking anxiety by neuroticism. Neuroticism was found to have a positive and significant impact on speaking anxiety (β = 0.33, *p* < 0.001). The model is significant, *F*(1, 284) = 35.88, *p* < 0.001, and explains 11% of the total variance (*R*^2^ = 0.11).

**Table 9:** Regression coefficients, standard errors and significance tests for the regression model.

Predictor	Coeff.	*SE*	*p*	*F*	*R^2^*
Constant	15.84	1.98	< 0.001		
Neuroticism	0.33	0.32	< 0.001	35.88	0.11

Table 10 presents the findings from the mediation analysis. Neuroticism significantly predicts anxiety sensitivity, and this relationship is positive (*a* = 0.21, *p* < 0.001). Anxiety sensitivity is positively and significantly related to speaking anxiety (*b* = 0.53, *p* < 0.001). The direct relationship between neuroticism and speaking anxiety (*c*) is significant in the first model (β = 0.33, *p* < 0.001), but the effect size decreases when anxiety sensitivity is added to the model (c′ = 0.22, *p* < 0.001). This decrease supports the partial mediating effect.

**Table 10:** The mediating role of anxiety sensitivity between neuroticism and speaking anxiety.

Model	Anxiety sensitivity (*M*)			Speaking anxiety (Y)		
	β	SE	*t*	β	SE	*t*
Neuroticism (X) *a*	0.21	0.40	3.68	*c'* 0.22	0.27	4.63
Anxiety Sensitivity (*M*)	–	–	–	*b* 0.53	0.04	11.19
Constant	17.94	2.48	7.23	7.90	1.80	4.39
	*R*^2^ = 0.05, *F*_(1, 284)_ = 13.54, *p* < 0.001			*R*^2^ = 0.38, *F*_(2, 283)_ = 88.45, *p* < 0.001		
**Bootstrap results for indirect effects**	β			**Boot SE**	**BootLLCI**	**BootULCI**
Indirect effect of anxiety sensitivity	0.11			0.03	0.05	0.18

**Figure 5:**
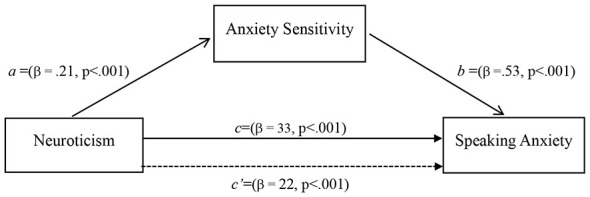
The mediating model of anxiety sensitivity in the relationship between neuroticism and speaking anxiety.

The bootstrap analysis revealed that neuroticism had a statistically significant indirect effect on speaking anxiety (β = 0.11, Boot SE = 0.03, 95% CI [0.05, 0.18]). The confidence interval (CI) does not include zero, indicating that the mediating effect is significant. Figure 5 summarizes the partial mediation model obtained.

Table 11 presents the results for predicting speaking anxiety from openness to experience. Openness to experience was found to have a negative and significant impact on speaking anxiety (β = −0.45, *p* < 0.001). The model is significant, *F*(1, 284) = 72.81, *p* < 0.001, and explains 20% of the total variance (*R*^2^ = 0.20).

**Table 11:** Regression coefficients, standard errors and significance tests for the regression model.

Predictor	Coeff.	*SE*	*p*	*F*	*R^2^*
Constant	44.00	2.08	< 0.001		
Openness to experience	−0.45	0.30	< 0.001	72.81	0.20

Table 12 presents the findings from the mediation analysis. Openness to experience significantly predicts anxiety sensitivity, and this relationship is negative (*a* = −0.33, *p* < 0.001). Anxiety sensitivity is positively and significantly related to speaking anxiety (*b* = 0.49, *p* < 0.001). The direct relationship between openness to experience and speaking anxiety (c) is significant in the first model (β = −0.45, *p* < 0.001), but the effect size decreases when anxiety sensitivity is added to the model (*c*′ = −0.29, *p* < 0.001). This decrease supports the partial mediating effect.

**Table 12:** The mediating role of anxiety sensitivity between openness to experience and speaking anxiety.

Model	Anxiety Sensitivity (*M*)			Speaking Anxiety (Y)		
	β	SE	*t*	β	SE	*t*
Openness to Experience *a* (X)	−0.33	0.38	−5.82	*c'* −0.29	0.27	−6.09
Anxiety Sensitivity (*M*)	–	–	–	*b* 0.49	0.04	10.10
Constant	41.35	2.66	15.53	27.37	2.43	11.26
	*R*^2^ = 0.11, *F*_(1, 284)_ = 33.87, *p* < 0.001			*R*^2^ = 0.41, *F*_(2, 283)_ = 100.32, *p* < 0.001		
**Bootstrap results for indirect effects**	β			**Boot SE**	**BootLLCI**	**BootULCI**
Indirect effect of anxiety sensitivity	−0.16			0.03	−0.23	−0.09

**Figure 6:**
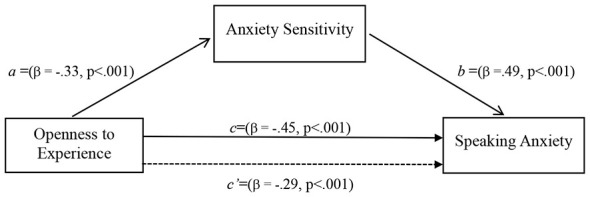
The mediating model of anxiety sensitivity in the relationship between openness to experience and speaking anxiety.

The bootstrap analysis revealed that openness to experience had a statistically significant indirect effect on speaking anxiety (β = −0.16, Boot SE = 0.03, 95% CI [−0.23, −0.09]). The confidence interval does not include zero, indicating that the partial mediating effect is significant. Figure 6 summarizes the obtained mediation model.

## Discussion

The study found significant correlations between all sub-dimensions of the Five-Factor Model of Personality and L2 speaking anxiety. This finding suggests that speaking anxiety can be considered not only in relation to language skills or the learning environment, but also in conjunction with individual characteristics. In particular, speaking in a second language is a process in which the individual expresses themselves, and the likelihood of being evaluated becomes more apparent. Therefore, individuals' communication styles, emotional tendencies, and reactions to performance situations may be related to the speaking experience (Akaraphattanawong et al., 2024; Zheng et al., 2024). This study's findings demonstrate that speaking anxiety among international students learning Turkish as a second language can be assessed alongside individual characteristics. Recent studies also indicate that foreign language speaking anxiety is a multifactorial construct shaped by personality, self-efficacy, enjoyment, emotional variables, and classroom-based interactional experiences rather than by linguistic competence alone (Babanoglu, 2025; Hussain et al., 2025).

In the study, a significant negative relationship was found between extraversion and L2 speaking anxiety. The literature often reports that individuals with high extraversion experience lower speaking anxiety. It has been reported that extraverted individuals exhibit higher self-confidence in social interactions and greater resilience in evaluative situations. These traits are suggested to have a negative relationship with speaking anxiety in social performance contexts. Indeed, evidence shows that extraversion is related to foreign language speaking anxiety and communication self-efficacy (Kabir et al., 2022). Additionally, extraversion is negatively associated with test anxiety, evaluation anxiety, and speaking anxiety (Erzhanova et al., 2024). These findings support the link between extraversion and speaking anxiety in terms of social self-confidence and perceived evaluation.

In this study, a significant negative relationship was found between agreeableness and L2 speaking anxiety. It is understood that people with high agreeableness tend to exhibit more developed empathy, cooperation, and social adaptability. However, the link between this personality trait and speaking anxiety is not straightforward or one-dimensional. Sometimes, a tendency toward social conformity and acceptance helps individuals feel more comfortable, but in other cases, an excessive need for approval and anticipation of negative evaluation can lead to higher anxiety levels. One study reported that agreeableness was positively related to speaking anxiety, especially when considering sensitivity to social expectations (Ünal and Pamuk, 2025). Conversely, some research indicates that individuals with high agreeableness actually experience lower communication anxiety and less stress during social interactions (Erzhanova et al., 2024). It is conceivable that this discrepancy may stem from the samples and learning contexts used in the studies (Chen et al., 2022). In particular, when speaking a second language, individuals may face not only the need to communicate but also the possibility of being evaluated and making mistakes. This situation may have contributed to the fact that the relationship between the adaptability trait and speaking anxiety has not consistently emerged in the same direction across different studies. Furthermore, variations in expectations regarding interpersonal relationships and social adaptation across cultures may be considered alongside differences in findings (Gaitán-Aguilar et al., 2022; Tang and Zhang, 2023; Zhang and Ting, 2024). Similarly, another study found that when resilience and self-efficacy were taken into account, agreeableness was associated with lower anxiety levels (Cassaretto et al., 2024). Additionally, agreeableness is associated with perceived social support, which, in turn, is linked to psychological distress (Shu et al., 2022). This interpretation is also supported by recent findings indicating that personality traits may indirectly influence foreign language anxiety through self-efficacy and interpersonal-emotional adjustment processes (Liu et al., 2026; Qin and Li, 2024).

The results indicated a significant and negative relationship between the conscientiousness sub-dimension and L2 speaking anxiety. The literature reports that people with high levels of conscientiousness tend to report lower levels of speaking anxiety (Hussain et al., 2022). Conscientiousness is associated with a tendency to behave in an orderly, planned, disciplined, and goal-oriented manner (Turner and Hodis, 2025). It is stated that these traits are related to individuals' levels of preparation and perceived sense of control in performance-demanding situations and, therefore, may be accompanied by lower perceived uncertainty (Ringwald et al., 2024). Indeed, it is emphasized that conscientiousness should be considered in an indirect, relational context alongside anxiety variables. It has been reported that when conscientiousness is evaluated alongside variables such as self-efficacy and academic burnout, it exhibits negative relationships with general anxiety levels (Wu et al., 2024). A study conducted with teacher candidates also found that individuals with high conscientiousness reported lower speaking anxiety, consistent with the importance they attached to preparation and performance control (Ünal and Pamuk, 2025).

Another finding of the study revealed a positive and significant relationship between neuroticism and speaking anxiety. The literature frequently reports that neuroticism is a personality dimension strongly associated with speaking anxiety. It is stated that neurotic individuals are more prone to negative cognitive evaluations in situations that are open to evaluation and stressful, have higher threat perceptions, and exhibit anxiety-prone moods. These characteristics are considered together with the cognitive, emotional, and physiological components of speaking anxiety. In a study conducted with teacher candidates, it was reported that individuals with high levels of neuroticism had higher levels of speaking anxiety (Ünal and Pamuk, 2025). Similarly, neuroticism has been shown to have positive correlations with test anxiety, communication anxiety, and fear of negative evaluation (Erzhanova et al., 2024). Furthermore, neuroticism is positively related to general anxiety and psychological distress levels, whereas personality traits such as extraversion, openness, and conscientiousness show negative relationships with anxiety indicators (Shu et al., 2022; Wu et al., 2024). This finding is consistent with recent literature indicating that anxiety-related personality tendencies are closely associated with foreign language anxiety, particularly when learners perceive communicative situations as evaluative or threatening (Babanoglu, 2025; Hussain et al., 2025).

The present study found a significant and negative relationship between openness to experience and speaking anxiety. “Openness to experience” is a personality trait characterized by cognitive flexibility, creativity, and a tendency to adapt to new situations. It is reported that these traits are associated with individuals' emotional responses in open-to-evaluation communication situations. The literature reports negative relationships between openness to experience and speech and communication anxiety. Indeed, it has been shown that levels of openness to experience are inversely related to test anxiety, communication anxiety, and fear of negative evaluation (Erzhanova et al., 2024). Similarly, openness to experience has been reported to be negatively related to general anxiety levels and positively related to self-efficacy and psychological resilience (Wu et al., 2024; Shu et al., 2022). Furthermore, openness to experience has been reported to be positively associated with second-language speaking self-efficacy and negatively associated with speaking anxiety (Kabir et al., 2022). Similarly, studies on EFL learners suggest that openness-related qualities such as flexibility, willingness to communicate, and mindfulness may help reduce speaking anxiety by supporting more adaptive responses to language-learning challenges (Ersanli and Ünal, 2022; Hussain et al., 2025).

This study found that anxiety sensitivity plays a mediating role in the relationship between the extraversion subscale of the “Five-Factor Personality Model” and speaking anxiety. Anxiety sensitivity refers to an individual's tendency to perceive physical and cognitive symptoms related to anxiety as threatening. The literature reports that individuals with low extraversion levels report higher anxiety sensitivity and that this trait is associated with speaking anxiety (Shu et al., 2022; Wu et al., 2024). In this context, it is stated that individuals with high levels of extraversion, who have lower anxiety sensitivity, exhibit negative correlations with speaking anxiety indicators. Similarly, studies on social and performance anxiety support anxiety sensitivity as a significant mediating variable in the relationship between extraversion and anxiety (Cassaretto et al., 2024).

In the study, anxiety sensitivity was found to play a mediating role in the relationship between agreeableness level and speaking anxiety. The literature reports that individuals with high agreeableness levels have more developed emotion regulation skills and lower anxiety sensitivity. It is stated that these individuals interpret physiological anxiety symptoms as less threatening and that this situation exhibits negative relationships with speaking anxiety (Shu et al., 2022; Wu et al., 2024). Indeed, it has been reported that when resilience is considered alongside emotional stability, it shows indirect relationships with anxiety indicators (Cassaretto et al., 2024). Similarly, it has been shown that agreeableness is negatively associated with fear of evaluation and communication anxiety, and that this relationship is linked to the regulation of emotional responses (Erzhanova et al., 2024). Conversely, it is stated that individuals with low levels of agreeableness report higher anxiety sensitivity and exhibit higher speaking anxiety in evaluation-sensitive communication contexts. These findings suggest that the relationship between speaking anxiety and agreeableness can be considered in the context of anxiety sensitivity.

According to another finding of the study, anxiety sensitivity plays a mediating role in the relationship between conscientiousness level and speaking anxiety. The literature reports that people with high levels of conscientiousness report lower anxiety sensitivity and perceive anxiety symptoms as less threatening (Shu et al., 2022; Wu et al., 2024). It is stated that these individuals approach stressful situations in a more structured way and have more developed self-control and emotion regulation skills. Indeed, studies that indirectly examine the relationship between conscientiousness and anxiety indicators show that when this personality dimension is evaluated alongside resilience, self-efficacy, and cognitive control variables, it exhibits negative relationships with anxiety sensitivity (Cassaretto et al., 2024; Wu et al., 2024). Furthermore, it has been reported that the level of conscientiousness is negatively related to general anxiety symptoms (Shu et al., 2022).

This study found that anxiety sensitivity plays a mediating role in the relationship between neuroticism and speaking anxiety. The literature reports that people with high levels of neuroticism perceive anxiety symptoms more intensely and threateningly, which in turn shows a positive relationship with speaking anxiety (Shu et al., 2022; Wu et al., 2024). Indeed, neuroticism is positively related to test anxiety, communication anxiety, and fear of negative evaluation (Ünal and Pamuk, 2025; Erzhanova et al., 2024). Furthermore, it has been argued that high levels of neuroticism are associated with negative interpretations of physiological arousal symptoms and increased anxiety sensitivity (Cassaretto et al., 2024).

This study found that anxiety sensitivity plays a mediating role in the relationship between openness to experience and speaking anxiety. The literature reports that individuals with high openness to experience perceive anxiety symptoms as less threatening, along with cognitive flexibility, curiosity, and adaptability tendencies. It is stated that these individuals evaluate anxiety symptoms as temporary and part of the learning process and, therefore, have lower anxiety sensitivity (Erzhanova et al., 2024; Shu et al., 2022). Indeed, it has been shown that “openness to experience” is negatively related to general anxiety levels, and this relationship is indirectly shaped when considered alongside variables such as self-efficacy, emotion regulation, and anxiety sensitivity (Wu et al., 2024). Similarly, openness is significantly associated with lower anxiety indicators, as well as with resilience and emotional flexibility (Cassaretto et al., 2024). Furthermore, a study on second language speaking anxiety reported that openness to experience was positively related to communication self-efficacy and negatively related to speaking anxiety (Kabir et al., 2022).

When all the findings are considered as a whole, it appears that anxiety sensitivity follows a similar pattern in the relationships between personality traits and L2 speaking anxiety. This result suggests that individuals may differ not only in terms of their personality traits but also in terms of the meaning they attach to anxiety symptoms and how they evaluate these symptoms. Particularly when considering situations such as the expectation of making mistakes, not being understood, or receiving negative evaluations during the process of speaking a second language, it appears meaningful to assess anxiety sensitivity in conjunction with speaking experience. In this context, it is thought that taking anxiety sensitivity into account alongside personality traits in studies addressing L2 speaking anxiety could contribute to more comprehensive interpretations. Taken together, the current findings are consistent with recent studies suggesting that L2 speaking anxiety should be interpreted as a multidimensional psychological experience shaped by personality traits, anxiety-related sensitivity, self-efficacy, resilience, fear of negative evaluation, mindfulness, and emotional factors rather than as a purely linguistic difficulty (Babanoglu, 2025; Düzler and Ekizer, 2025; Ersanli and Ünal, 2022; Hussain et al., 2025; Liu et al., 2026; Qin and Li, 2024).

### Limitations and future directions

From the standpoint of external validity and generalizability (Cook and Campbell, 1979; Shadish et al., 2002), several limitations should be considered when interpreting the present findings. The participants consisted of adult international students within a relatively narrow age range and educational context. Such a relatively homogeneous sample may limit the generalisability of the findings, as foreign language anxiety varies with learner characteristics and learning contexts (Babanoglu, 2025; Hussain et al., 2025). In addition, although both male and female students participated in the study, the predominance of male participants suggests that the findings should be interpreted cautiously when extending them to other gender groups. Future studies including more balanced samples with respect to gender, age, profession, and cultural background would provide a broader understanding of how personality traits relate to Turkish L2 speaking anxiety across diverse populations (Liu et al., 2026).

From the standpoint of correlational inference and cross-sectional mediation, caution is also warranted (Shadish et al., 2002; Maxwell and Cole, 2007). The cross-sectional nature of the study limits the interpretation of the findings. Because all variables were measured at a single point in time, the observed relationships can only be interpreted as correlational rather than causal. Recent research has continued to emphasize the importance of longitudinal and experimental designs for understanding the developmental nature of foreign language anxiety and the psychological mechanisms underlying changes in language learning experiences (Alamer et al., 2025). Future studies employing longitudinal or experimental methodologies would therefore provide stronger evidence regarding the mediating role of anxiety sensitivity.

From a measurement perspective, classic work on construct validity has long emphasized that the adequacy of inferences depends on how well the instruments capture the intended constructs (Cronbach and Meehl, 1955; Messick, 1989). Another limitation concerns the assessment of Turkish language proficiency. Although participants were enrolled in Turkish-language programs, proficiency was not measured with an independent, standardized instrument. Considering that language proficiency has been consistently linked to self-efficacy and foreign language anxiety, incorporating objective proficiency measures would strengthen the interpretation of future findings (Düzler and Ekizer, 2025; Qin and Li, 2024). The assessment of personality traits was based on the short form of the Big Five Personality Scale. While short-form measures reduce administration time and participant burden, they may not fully capture the complexity of personality dimensions. Recent research continues to recommend the use of more comprehensive personality assessments when examining the psychological mechanisms underlying anxiety and language learning (Qin and Li, 2024). Employing longer personality inventories in future studies may therefore provide a more nuanced understanding of the specific personality facets associated with Turkish L2 speaking anxiety.

From the standpoint of procedural or method bias in survey research, the data-collection procedure also warrants caution (Podsakoff et al., 2003). Although Harman's one-factor test suggested that common-method variance was not dominant, the study relied on self-report instruments administered in a single online session and in a single language. This design may still have introduced shared method effects related to response tendencies, social desirability, or common scale format. The multicultural nature of the sample also deserves further consideration. Although participants came from different countries, their cultural background was not explicitly examined as a variable in the proposed model. Contemporary research suggests that language anxiety is shaped not only by individual characteristics but also by intercultural experiences, psychological resilience, and fear of negative evaluation in multilingual environments (Liu et al., 2026). Examining cultural values and testing the proposed model across different cultural contexts would therefore enhance our understanding of the cultural variability of Turkish L2 speaking anxiety. Finally, the present model focused primarily on personality traits and anxiety sensitivity. However, recent studies indicate that foreign language anxiety is influenced by a broader network of psychological variables, including self-efficacy, self-esteem, resilience, cognitive flexibility, and perceived social support (Babanoglu, 2025; Düzler and Ekizer, 2025; Qin and Li, 2024). Incorporating these variables into future models may provide a more comprehensive explanation of the psychological processes underlying Turkish L2 speaking anxiety.

## Conclusion

In this study, the relationship between the sub-dimensions of the “Big Five Personality Model” and L2 speaking anxiety, and the mediating role of anxiety sensitivity in this relationship, was examined. This study revealed that all dimensions of the “Five-Factor Personality Model” have significant relationships with L2 speaking anxiety. The findings reveal that personality traits are significantly associated with individuals' communication experiences and emotional responses. It could be argued that personality can be regarded as one of the psychological characteristics associated with individuals' experiences of anxiety during the speaking process. According to the results, there are significant negative relationships between extraversion, agreeableness, conscientiousness, openness to experience, and speaking anxiety. This finding suggests that lower levels of speaking anxiety are observed in individuals with higher levels of extraversion, conscientiousness, agreeableness, and openness to experience. Conversely, individuals with high neuroticism levels were found to have significantly higher speaking anxiety. This finding is consistent with the view that neuroticism is an important correlate of speaking anxiety, although fear of negative evaluation was not measured directly in the present study.

One of the most notable findings of the study is the mediating role of anxiety sensitivity. Analyses showed that anxiety sensitivity has a significant mediating effect on the relationships between all sub-dimensions of the “Five-Factor Personality Model” and speaking anxiety. In particular, individuals with high levels of agreeableness, extraversion, and conscientiousness, and low anxiety sensitivity, have been found to exhibit lower levels of L2 speaking anxiety. On the other hand, increased anxiety sensitivity has been observed alongside higher levels of L2 speaking anxiety in individuals with high levels of neuroticism. Similarly, in the openness to experience dimension, low anxiety sensitivity allows individuals to approach new learning and communication experiences more comfortably. In conclusion, the findings suggest that Big Five personality traits were associated with Turkish L2 speaking anxiety both directly and indirectly, with anxiety sensitivity showing a significant partial mediating role in these relationships. The findings highlight the importance of considering personality traits and affective factors in foreign language teaching.

## Data Availability

The raw data supporting the conclusions of this article will be made available by the authors, without undue reservation.
